# Where do you want your drugs delivered?

**DOI:** 10.1080/17453674.2019.1707951

**Published:** 2020-01-13

**Authors:** Ming Ding, Ivan Hvid

**Affiliations:** a Orthopaedic Research Unit, Department of Orthopaedic Surgery and Traumatology, Odense University Hospital, and Department of Clinical Research, University of Southern Denmark;; b Section of Children’s Orthopaedics and Reconstructive Surgery, Division of Orthopaedic Surgery, Oslo University Hospital

Promoting bone repair and regeneration in bone fractures by local delivery of growth factors possessing osteoinductive activity has been extensively investigated with significant advancements. Methods such as a combination of biomaterial-based scaffold and local bone active molecule delivery has been used (Raina et al. 2019b). Unfortunately, the local delivery approaches most often require surgery, and for some complex clinical fractures it is not possible to apply an invasive strategy because of access restrictions. Thus, to achieve efficient local concentrations and reduce systemic side effects, an ideal alternative method would be systemically administered targeted delivery of drugs due to ease of handling and precise spatiotemporal compatibility at fracture sites or sites of bone regeneration. However, the combination of delivery of osteopromotive molecules locally and systemically to enhance bone regeneration has been reported with limited success.

Recently, Raina and co-workers demonstrate a novel method using synthetic hydroxyapatite (HAP) particles as a recruiting moiety for different drug classes administered systemically, showing that their affinity to HAP binding sites can activate the particulate material to exert a biological effect (Raina et al. 2019a). Systemically administered biomolecules (zoledronic acid, tetracycline and 18F-fluorine) all sought the HAP moiety placed in a muscle pouch. Significantly higher peri-implant bone volume and peak force were observed around an implant containing HAP particles relative to an empty implant. It was found possible to reload HAP particles on as-needed basis. The uptake of the antibiotic tetracycline was observed in the biomaterial by fluorescence microscopy, and the uptake of the radioemitter ^18^F-fluorine was documented in the biomaterial by positron emission tomography/computed tomography (PET/CT). Thus, the targeted accretion in locally implanted particulate HAP is achieved by systemic drug administration, loading the biomaterial which is biologically activated (Raina et al. 2019a).

Importantly, several issues need to be controlled to achieve successful results. First, the targeted delivery of drugs should have a specific and high affinity to HAP binding sites, where micro- to nanoparticles of HAP at the site acting as both a carrier and a recruiting moiety for systemically administered drugs. Examples are bone-seeking drugs like bisphosphonates for bone regeneration, HAP binding antibiotics like tetracycline for infection (Perrin 1965), and bone-seeking radioactive isotopes like ^186^Re for metastatic bone disease (de Klerk et al. 1992). Tetracycline can also be used for dynamic histomorphometry of bone. Second, the timing of the injection may be critical. In rat models of fracture healing, the timing between 1 and 2 weeks of a single systemic dose of zoledronic acid plays an important role in the modulation of callus properties and for HAP deposition in a fracture callus collagen network, and fits well with the fluid mechanics necessary for drug transport (Amanat et al. 2007).

The current promising results using HAP as a recruiting and reloadable particulate apatite moiety to which systemically administered drugs circulating in the bloodstream could bind due to a high chemical affinity provides a novel method for treatment of various bone diseases. Apart from its promise for bone regeneration, it is also potentially applicable in the treatment of bone infections, tumors, and osteoporosis. *Staphylococcus aureus* for example is a causative agent of osteomyelitis and has a high affinity for bone, and can induce osteonecrosis and resorption of bone matrix (Lucke et al. 2003). A novel therapeutic approach may be by using nano- or micro-HA particles already functionalized with antibiotics, to provide extended sustained local antibiotic delivery during and after radical surgery, and later reloading HA particles on as-needed basis. A similar method can apply to metastatic bone disease by using radioactive isotopes and chemotherapeutics for treatment of locally malignant tumors or solitary metastasis (Raina et al. 2019a).

Since HAP is already abundant in bone, implantation of HAP would not necessarily be required. Several bone mineral seekers are known apart from bisphosphonates, tetracycline, and fluorine—certain peptides for example (Rotman et al. 2018). The challenge of the future would be to find bone-seeking molecules or nanoconstructs that can be systemically administered, can carry the appropriate drug or pro-drug, can circulate in the body without significant global effects, can bind to the bone site of interest (which might be the whole skeleton, e.g., in osteoporosis), and exert its action directly or by pro-drug activation. Certainly, much work needs to be performed before safe and efficient drug delivery systems to bone become useful on a larger scale.
